# Time-Series Analyses of Air Pollution and Mortality in the United States: A Subsampling Approach

**DOI:** 10.1289/ehp.1104507

**Published:** 2012-10-24

**Authors:** Suresh H. Moolgavkar, Roger O. McClellan, Anup Dewanji, Jay Turim, E. Georg Luebeck, Melanie Edwards

**Affiliations:** Exponent Inc., Bellevue, Washington, USA; Fred Hutchinson Cancer Research Center, Seattle, Washington, USA; Toxicology and Human Health Risk Analysis, Albuquerque, New Mexico, USA; Indian Statistical Institute, Kolkata, India

**Keywords:** criteria pollutants, hierarchical Bayes, multicity analyses, spline smoothers, subsampling bootstrap

## Abstract

Background: Hierarchical Bayesian methods have been used in previous papers to estimate national mean effects of air pollutants on daily deaths in time-series analyses.

Objectives: We obtained maximum likelihood estimates of the common national effects of the criteria pollutants on mortality based on time-series data from ≤ 108 metropolitan areas in the United States.

Methods: We used a subsampling bootstrap procedure to obtain the maximum likelihood estimates and confidence bounds for common national effects of the criteria pollutants, as measured by the percentage increase in daily mortality associated with a unit increase in daily 24-hr mean pollutant concentration on the previous day, while controlling for weather and temporal trends. We considered five pollutants [PM_10_, ozone (O_3_), carbon monoxide (CO), nitrogen dioxide (NO_2_), and sulfur dioxide (SO_2_)] in single- and multipollutant analyses. Flexible ambient concentration–response models for the pollutant effects were considered as well. We performed limited sensitivity analyses with different degrees of freedom for time trends.

Results: In single-pollutant models, we observed significant associations of daily deaths with all pollutants. The O_3_ coefficient was highly sensitive to the degree of smoothing of time trends. Among the gases, SO_2_ and NO_2_ were most strongly associated with mortality. The flexible ambient concentration–response curve for O_3_ showed evidence of nonlinearity and a threshold at about 30 ppb.

Conclusions: Differences between the results of our analyses and those reported from using the Bayesian approach suggest that estimates of the quantitative impact of pollutants depend on the choice of statistical approach, although results are not directly comparable because they are based on different data. In addition, the estimate of the O_3_-mortality coefficient depends on the amount of smoothing of time trends.

Time-series analyses of air pollution and various health end points, including daily mortality, using flexible generalized linear models (GLMs) or generalized additive models (GAMs), have become commonplace in the last decade. The National Morbidity, Mortality and Air Pollution Study (NMMAPS) was an ambitious effort undertaken by scientists at Johns Hopkins University (Baltimore, MD, USA) and Harvard University (Cambridge, MA, USA) to investigate the association between particulate pollution (i.e., particulate matter with an aerodynamic diameter ≤ 10 µm; PM_10_) and morbidity and mortality in the 90 largest metropolitan areas in the United States over the period 1987–1994 using time-series methods (Dominici et al. 2003; Samet et al., 2000a, 2000b, 2000c). For the mortality analyses, the investigators used a common approach for analyses of the time-series data on daily PM_10_ levels and deaths for each city. Specifically, a national mean estimate for the association between PM_10_ and mortality was obtained by combining the individual city-specific estimates using a hierarchical Bayes procedure, which assumed that the city-specific effects were normally distributed. Since then the analyses have been extended to include more cities, more years of data, and a second pollutant, ozone (O_3_) (Bell et al. 2004; Smith et al. 2009). In this article, we propose an approach to analyzing multicity time-series data that is complementary to the hierarchical Bayes approach.

Arguably, for standard setting, one might want to estimate not a national mean effect, but a common pollutant effect across the country. The most direct way to estimate a common pollutant effect on mortality across multiple cities would be to analyze all cities simultaneously using a GLM or GAM model postulating a common pollutant effect estimate across cities, but with control of confounders specific to each city. A statistical test for the hypothesis that there is a common pollutant effect across cities could then be based on standard likelihood-based procedures or on the Akaike or Bayes information criterion (AIC or BIC). Alternatively, a computational approach to investigating the distribution of a common estimator of a pollutant effect is to use the bootstrap or the jackknife procedure on the full complement of cities (Efron and Tibshirani 1993). However, with a large number of cities this conceptually simple approach presents formidable computational problems. One way around the computational problems is to use either the “delete-*k* jackknife” followed by the bootstrap procedure (Efron and Tibshirani 1993) or the subsampling procedure (Politis et al. 1999). In this study, we used the subsampling procedure to analyze the time-series data on all the criteria pollutants, with the exception of lead, and mortality in 108 metropolitan areas in the United States over the 14-year period 1987–2000.

## Methods

We downloaded the mortality and air pollution time-series data from the NMMAPS web site maintained by the Johns Hopkins investigators (http://www.ihapss.jhsph.edu/data/data.htm). Using the same database allowed direct comparisons to be made to the previous work of the Johns Hopkins team and others using these data. Daily data on the number of deaths are available for 108 metropolitan areas over the 14-year period 1987–2000. Daily concentrations of the criteria pollutants—PM_10_, O_3_, carbon monoxide (CO), nitrogen dioxide (NO_2_), and sulfur dioxide (SO_2_)—are also available; although for each of the pollutants, information is available only for a subset of the days. A limitation, shared with all epidemiologic analyses of air pollution data, is that the data were collected from stationary monitors to determine compliance with air quality regulation. Thus, the data collected are not necessarily ideal for epidemiologic studies. We recognize also that ambient concentrations are imperfect surrogates for personal exposure.

For the usual bootstrap approach, for each bootstrap cycle, 108 cities would be chosen with replacement from the original set of 108 cities, and the maximum likelihood estimate (MLE) of the pollutant effect computed using Poisson regression methods. This procedure would be repeated many times to obtain an estimate of the distribution of the common pollutant effect. However, with the large number of cities considered in these analyses, it is computationally infeasible to derive an MLE using this approach. Therefore, an alternative procedure, such as the delete-*k* jackknife (Efron and Tibshirani 1993) or the closely related subsampling procedure (Politis et al. 1999) must be implemented. For the delete-*k* jackknife followed by the bootstrap procedure, first the set of all possible subsets of size *d* = 108 – *k* is constructed from the 108 cities. Each bootstrap cycle then randomly selects a city from this set, and the common pollutant effect is estimated for the specific chosen set of *d* cities.

We used a closely related method, the subsampling procedure described by Politis et al. (1999). In this procedure, for each bootstrap cycle, we randomly chose *d* cities without replacement out of the 108 cities with available data, and estimated the common pollutant effect for each sample of *d* cities. Politis et al. (1999) recommend that *d* be much smaller than 108, and we chose *d* = 4. The choice of *d* is arbitrary; however, the confidence intervals (CIs) for the parameter estimates have to be adjusted for this choice as discussed below. The distribution of the estimator of the common pollutant effect was based on 5,000 bootstrap cycles.

Let *X*_1_, *X*_2_, …, *X_n_* be a sequence of independent observations (i.e., a sequence of realizations of independent random variables). In our case, each observation in the sequence represents the data on daily deaths, pollutant concentrations, and weather variables over the period 1987–2000 in each of the *n* cities considered in the analyses. The total number of cities, *n*, depends on the pollutant or combination of pollutants considered. For example, *n* = 102 for analyses that involve PM_10_ alone, and *n* = 56 for analyses that involve all pollutants. Let Θ be the parameter representing the common effect of a pollutant, and let Θ*_n_* be the MLE for Θ based on *n* observations and Θ*_d_* the MLE for Θ based on *d<<n* observations (i.e., based on a subset of *d* cities), then, by general maximum likelihood theory, Θ*_n_* and Θ*_d_* converge to Θ with rates _√_*n* and _√_*d*, respectively.

Under standard likelihood-based procedures, inferences would be based on the distribution of Θ*_n_*. However, computation of Θ*_n_* and its distribution is infeasible with large *n*. Therefore, we based our inferences on Θ*_d_*, where in our case, *d* = 4, and based our CIs on the subsampling distribution as described by Politis et al. (1999). The properties of the subsampling procedure hold under rather weak conditions. The sequence of observations is required to satisfy the α-mixing condition (Politis et al. 1999, p. 315). This condition is seen to be trivially satisfied by a sequence of independent observations. An anonymous referee suggested that sampling units other than entire cities might be more appropriate. We agree that other sampling schemes need to be explored.

CIs were computed after adjusting for the size of the subsample as follows. If F_d_* represents the empirical distribution function of _√_*d* (Θ*_d_* – Θ), then for any significance level α,

F_d_^*–1^(α/2) < _√_*n* (Θ*_n_* – Θ) < F_d_^*–1^(1 – α/2), [1]

with probability close to 1 – α. It then follows that Θ*_n_* – (1/_√_*n*)F_d_^*–1^(1 – α/2) < Θ < Θ*_n_* – (1/_√_*n*)F_d_^*–1^(α/2) is a 1 – α CI for Θ. In our case, we were unable to estimate Θ_n_ because of computational issues. We therefore approximated Θ*_n_* by (ΣΘ*_d_*)/*N*, where *N* = 5,000, the total number of subsamples drawn. That is, we approximated Θ*_n_* as the mean of the Θ*_d_*.

Thus, for our analyses, for each bootstrap cycle, we drew a random sample of four cities without replacement from among the 108 cities with available data. We then fit an over-dispersed Poisson model to the randomly chosen 4 cities to obtain the MLE of the common pollutant effects on mortality in the 4 cities, but with confounders, such as temperature and relative humidity, being separately controlled in each of the 4 cities. Because a number of previous analyses (e.g., Bell et al. 2004; Dominici et al. 2003) have considered the effect of the pollutants with a 1-day lag, we have done the same in these analyses. Likewise, the number of degrees of freedom (df) for time trends and weather are also consistent with those used in previous analyses (Bell et al. 2004; Dominici et al. 2003; Moolgavkar 2003). Specifically, for each bootstrap cycle we modeled the number of deaths from all causes (with accidents and suicides removed) in a city on a specific day as a function of the 24-hr average pollutant concentration on the previous day, temporal trends (50 or 100 df natural spline), day of the week (categorical variable), mean temperature on the previous day (6 df natural spline), and mean dew-point temperature on the previous day (6 df natural spline). Note that this model controlled for confounders, day of week effects, and time trends in a city-specific fashion.

To investigate the shape of the ambient concentration–response relationship, we used the same models with the pollutant effects represented by natural splines with 6 df. Multiple pollutants that have been concurrently measured can be easily added as covariates in these analyses. Because of missing data, the size of the data set from which samples of cities were drawn for analyses depended on the number of pollutants considered.

Clearly, the choice of lags for specific pollutants and for the weather variables—temperature, and relative humidity—should be based on biological considerations whenever possible. Unfortunately, there is little information to guide these choices. In previous publications, a 1-day lag has often been used. For example, in the revised NMMAPS analyses, Dominici et al. (2003) used the same lag for each of the pollutants in their multipollutant analyses. Their results indicate that a 1-day lag yields close to the maximum impact on daily mortality. It would be possible also to consider other lag structures, such as distributed lag models. However, the purpose of this study was not to be a comprehensive reanalysis of the NMMAPS data, but to provide an approach to the analysis of national data that complements the Bayesian approach. Similarly for the weather covariates, we used a 1-day lag because there is little biological information to suggest that any specific lag structure is better than any other.

The model was fit to the data using the R software package (http://www.r-project.org). The means of the 5,000 maximum likelihood estimates of the common pollutant effects were approximately unbiased and consistent estimators of the common national effects of the pollutants.

We conducted simulations to investigate the coverage properties of the CIs constructed as described above. Specifically, we generated 100 observations based on a Poisson variate with an intercept and a slope. From these 100 observations, we drew 5,000 subsamples of size 4 without replacement and computed 90% and 95% CIs as described above in addition to computing the usual likelihood-based CIs. We repeated this entire procedure 1,000 times to investigate the coverage properties of the CIs. We found that the mean of the 5,000 subsample estimates was an excellent approximation to the MLE (data not shown). While the usual likelihood-based CIs covered the true values of the parameters with the nominal coverage probabilities, the subsampling CIs were conservative, that is, their coverage probabilities were larger than the nominal coverage probabilities. The 95% CIs covered the true values of the parameters approximately 98% of the time and the 90% CIs covered the true values of the parameters approximately 95% of the time (data not shown). Therefore, because the estimated CIs were highly conservative (i.e., too wide), we present both 95% and 90% CIs in our tables in the present study and note that our tests of significance (i.e., whether the CIs contain 0) are also conservative (i.e., the actual level of significance is smaller than the nominal alpha). The coverage properties of both of the CIs constructed using the subsampling approach and the credible intervals constructed in the hierarchical Bayes approach need to be explored in realistic simulation scenarios.

## Results

Bootstrap means and 90% and 95% CIs after small-sample corrections for common nationwide estimates of effects of pollutants represent associations with incremental changes in the 24-hr average concentration on the previous day of 10 μg/m^3^ for PM_10_; 10 ppb for O_3_, SO_2_, and NO_2_; and 1 ppm for CO. The unit measures for the individual pollutants were chosen to facilitate comparisons with previous estimates rather than typical day-to-day variations in ambient concentrations, which vary among cities. With time trends smoothed using 100 df natural splines, all pollutants were significantly (at the 0.05 level) associated with mortality in single-pollutant models (Table 1). With 50 df natural splines for time trends, the estimated coefficient for O_3_ was greatly attenuated and statistically insignificant.

**Table 1:** Estimated mean percent change in daily mortality associated with a unit increase in pollutant concentration on the previous day, single-pollutant model analyses.

Pollutant	dfa	Mean	90% CI	95% CI	No. of cities
PM10		50		0.40		0.33, 0.51		0.30, 0.53		102
		100		0.39		0.30, 0.48		0.28, 0.49		
O3		50		0.08		–0.11, 0.34		–0.16, 0.38		98
		100		0.40		0.29, 0.53		0.27, 0.56		
SO2		50		1.60		1.14, 1.91		0.93, 1.94		85
		100		1.46		1.17, 1.70		1.07, 1.74		
NO2		50		1.01		0.91, 1.13		0.89, 1.16		72
		100		1.03		0.92, 1.14		0.91, 1.18		
CO		50		1.47		1.18, 1.71		1.15, 1.75		95
		100		1.30		1.09, 1.53		1.05, 1.58		
Units are 10 µg/m3 for PM10; 10 ppb for NO2, O3, and SO2; and 1 ppm for CO. Temperature and relative humidity on the previous day are controlled using 6 df natural splines. Time trends are controlled using either 50 df or 100 df natural splines. Day of week is controlled as a categorical variable. The last column shows the number of cities available for analyses. aDegrees of freedom for natural splines of time trends.										

The magnitudes of associations with incremental increases in CO, NO_2_, and SO_2_ were greater than for associations with PM_10_ and O_3_. When CO, NO_2_, and SO_2_ were included in the same model with 100 df splines for time trends, all three associations were attenuated and the association of CO with mortality was no longer statistically significant (Table 2).

**Table 2:** Estimated mean percent change in daily mortality associated with a unit increase in pollutant concentration on the previous day, three-pollutant model.

Pollutant	Mean	90% CI	95% CI
SO2		0.82		0.57, 1.08		0.48, 1.15
NO2		0.62		0.43, 0.92		0.40, 0.98
CO		0.64		–0.10, 1.06		–0.20, 1.09
Units are 10 ppb for NO2 and SO2 and 1 ppm for CO. Temperature and relative humidity on the previous day are controlled using 6 df natural splines; time trends are controlled using 100 df natural splines and day of week is controlled as a categorical variable. These joint-pollutant analyses are based on data from 58 cities.						

Two-pollutant analyses of PM_10_ and each of the gases with 100 df splines for time trends indicated significant associations for PM_10_ in all four models, although estimated effects were attenuated except in the model adjusted for O_3_ (Table 3). Effect estimates for CO, NO_2_, and SO_2_ were significant and consistent with estimates that were not adjusted for PM_10_, but the estimated effect of O_3_ on mortality was attenuated and was not statistically significant based on the 95% CI or even the 90% CI (Table 3).

**Table 3:** Estimated mean percent change in daily mortality associated with a unit increase in pollutant concentration on the previous day, two-pollutant model.

Pollutant	Mean	90% CI	95% CI
PM10		0.29		0.16, 0.42		0.13, 0.45
CO		1.23		0.68, 1.64		0.55, 1.70
PM10		0.20		0.07, 0.33		0.03, 0.36
NO2		0.94		0.66, 1.20		0.60, 1.26
PM10		0.33		0.23, 0.45		0.19, 0.46
SO2		1.33		0.66, 1.85		0.38, 1.97
PM10		0.39		0.29, 0.49		0.25, 0.51
O3		0.22		–0.0008, 0.43		–0.05, 0.48
Units are 10 µg/m3 for PM10; 10 ppb for NO2, O3, and SO2; and 1 ppm for CO. Temperature and relative humidity on the previous day are controlled using 6 df natural splines. Time trends are controlled using 100 df natural splines, and day of week is controlled as a categorical variable. These two-pollutant analyses are based on 92 cities for PM10 and CO, 72 cities for PM10 and NO2, 83 cities for PM10 and SO2, 95 cities for PM10 and O3.						

**Figure 1:**
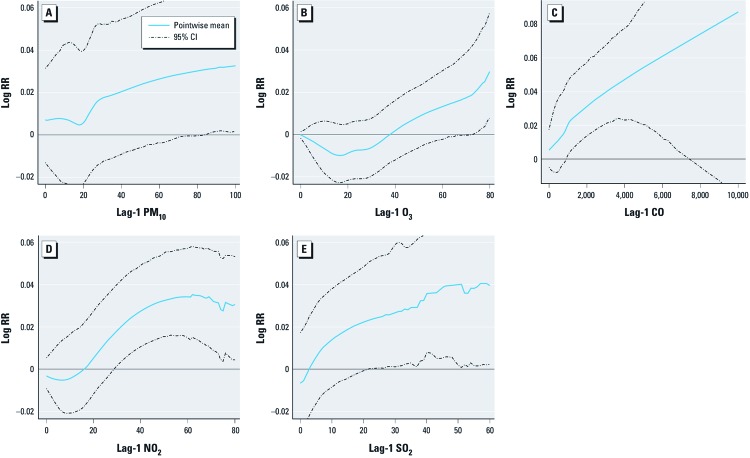
Flexible ambient concentration–response relationship between pollutants and deaths on the following day. Pointwise means and 95% CIs adjusted for size of the bootstrap sample (*d* = 4) as described in the text; RR, relative risk. (*A*) PM_10_, (*B*) O_3_, (*C*) CO, (*D*) NO_2_, (*E*) SO_2_.

Bootstrap means for flexible ambient concentration–response relationships, using 6 df natural splines, between pollutant concentrations and deaths on the following day estimated from single-pollutant models suggest nonlinearity and threshold-like behavior for NO_2_, PM_10_, and O_3_ (Figure 1). However, the CIs for NO_2_ and PM_10_ are wide and the concentration–response relationships are consistent with linearity.

Our approach assumes a common national pollutant effect. However, if there is a common national pollutant effect, then for a randomly chosen set of four cities, one would expect a common shared-effects model to have a lower AIC than a model postulating separate effects. Therefore, for each bootstrap cycle, we computed the AIC for the common pollutant effect model and for the individual models of each sample of four cities selected for each cycle. For all pollutants, the AIC was lower for the separate four-city fits than for the model postulating common pollutant effects for more than half the cycles (data not shown).

## Discussion

In this study, we used a subsampling bootstrap approach to estimating maximum likelihood estimates for common national effects for associations of individual pollutants with mortality. Previous analyses of NMMAPS data have used a hierarchical Bayes approach (e.g., Bell et al. 2004; Samet et al. 2000a, 2000b, 2000c; Smith et al. 2009) to estimate a national mean effect.

The procedure described in this study is one approach to estimating common pollutant effects, if they exist; however, in a vast geographically and climatically diverse country such as the United States, it is not unreasonable to expect heterogeneity of pollutant effects across the country. First, PM is a complex mixture whose composition varies by region and season. O_3_ could also be considered a mixture because it is generally present with other oxidants. Second, for all pollutants, any effects on human health would be expected to be modified by weather and by the circumstances of exposure, which clearly vary by region and season. A comparison of AIC statistics for sample-specific (four city) models and for common estimate models suggests that the four-city models fit the data better than the common model in most cases, which is consistent with heterogeneous effects among cities. This finding is consistent with the results of the hierarchical Bayes multicity analyses (Bell and Dominici 2008; Dominici et al. 2003; Smith et al. 2009). The heterogeneity of pollutant effects suggests that any single national estimate may not provide a reliable measure of the health benefits that would accrue from a reduction in pollutant concentrations.

An advantage of the Bayesian approach is that it allows for heterogeneity of city-specific coefficients, albeit with the simplifying assumptions that these are independent and identically distributed. That said, the hierarchical Bayes approach and the subsampling approach described in this study are complementary approaches to estimating a single national pollutant effect. Under both approaches, however, further investigation of heterogeneity requires that regional analyses be performed.

The use of Bayesian methods in multicity analyses was pioneered by the first investigators of NMMAPS (Dominici et al. 2000; Samet et al. 2000a, 2000b, 2000c). Their approach to the analyses of multicity data used a two-stage procedure, with analyses of single cities at the first stage, followed by a hierarchical Bayes analysis of the first-stage results to arrive at a single estimate of a national mean for the pollutant effect. Once the first-stage analyses were completed, the estimated pollutant effects were considered in isolation from the other covariates and combined in a second stage, resulting in a procedure that is operationally similar to a meta-analysis. This procedure assumes that the asymptotic distribution of the pollutant effect estimate has been achieved, and that a simple approximation to a full Bayesian analysis yields valid results in the second stage. The first assumption regarding the asymptotic distribution of parameter estimates is widely made in statistical procedures; in fact, one study (Dominici et al. 2000) reports that the asymptotic properties of the MLE are well approximated in the first-stage analyses. Our procedure also relied on asymptotic results.

The assumptions underlying the second-stage procedure may be more problematic. A fully Bayesian analysis would put prior distributions on all the parameters of the model, not just the pollutant coefficients, and allow the parameter estimates along with their covariance structure from the first stage to be carried forward to the second-stage analyses. Although this procedure may be possible in theory, it would be much more computationally intensive and may not be practical. Although it is not clear that a fully Bayesian analysis would make a substantial difference to the results, these issues do not arise with the approach proposed in this study, and common national pollutant–mortality coefficients and their CIs can be estimated in a single-stage analysis even when multiple pollutants are modeled using splines. However, the simple simulation that we performed indicated that the CIs generated using our approach were conservative. The coverage properties of CIs and credible intervals generated, respectively, by the subsampling and the hierarchical Bayes procedures need to be investigated using realistic and comprehensive simulation scenarios.

Smith et al. (2009) observed that the national mean from the hierarchical Bayes analysis approximates the mean (not the inverse variance weighted mean) of the individual city-specific estimates as the variance of assumed distribution of the city-specific coefficients goes to infinity. They cautioned against any use of a national statistic, but advocated the use of a population-weighted mean if a national statistic is computed. The mean of the distribution of the bootstrap samples we generated implicitly has a population weight built in because the common estimate for each bootstrap cycle is influenced by the sizes of the populations of the cities in that cycle.

Our CIs for the estimated common national effects of the pollutants are wider than the credible intervals reported in earlier studies using the hierarchical Bayes approach. This could be a consequence of hierarchical Bayes credible intervals that may be too narrow (because the fully Bayesian procedure is replaced by an approximation that ignores estimates of weather and time-trend parameters in the second-stage analyses), in addition to subsampling CIs that are too wide (conservative), as suggested by our simulations.

Previous multicity analyses have focused on PM_10_ or O_3_ (Bell et al. 2004, 2006; Dominici et al. 2003; Katsouyanni and Samet 2009; Smith et al. 2009). Analyses that addressed regional heterogeneity (Bell and Dominici 2008; Dominici et al. 2003; Smith et al. 2009) reported considerable heterogeneity of estimated coefficients for both PM and O_3_. Dominici et al. (2003) reported the largest PM_10_ coefficients were in the Northeast region among the seven regions they considered, followed by Southern California. The smallest PM_10_ coefficients were reported in the Upper Midwest. The estimated coefficients in the Northeast were twice as high as those reported for the Upper Midwest. Smith et al. (2009) estimated the highest O_3_ coefficients for the Northeast and the Industrial Midwest but did not estimate significant effects for Southern California, where levels of O_3_ have always been high. A similar gradient of O_3_ coefficients, with no associations in Los Angeles, intermediate effect estimates for Chicago, and larger effect estimates for New York have been reported in other studies (Moolgavkar 2003).

Previous time-series analyses of air pollution and mortality have focused on PM_10_ or O_3_, with other pollutants addressed as confounders, if at all (Dominici et al. 2003). Our findings suggest that the emphasis on PM and O_3_ may deserve reconsideration because estimated associations were strongest for CO, NO_2_, and SO_2_. In addition, coefficients for all three gases remained highly significant in joint pollutant analyses with PM_10_, and coefficients for NO_2_, and SO_2_ remained significant in a model that included all three gases.

*PM_10_*_._ The NMMAPS analyses estimated a national mean increase of 0.27% in mortality for a 10-μg/m^3^ increase in PM_10_ on the previous day using GAMs, and a 0.22% increase using a GLM. Our estimate of the mean with 50 df natural splines for time trends is 0.4% (95% CI: 0.30, 0.53). This estimate is not directly comparable to the estimates from the hierarchical Bayes analyses because we used somewhat different city-specific models and had many more cities with more years of data, although it is of interest to note that our estimate is virtually identical to the estimate reported for the Northeast (0.41; 95% posterior interval: 0.04, 0.78) in the NMMAPS reanalyses (Dominici et al. 2003).

In two-pollutant analyses of PM_10_ with CO, NO_2_, or SO_2_, the PM_10_ coefficients were somewhat attenuated but continued to be significantly associated with mortality. The association with PM_10_ was attenuated most in joint pollutant analyses with NO_2_. With the exception of the two-pollutant model of PM_10_ and O_3_, which produced a highly insignificant coefficient for O_3_ when a 50 df smoother was used, results were robust to the degree of smoothing of time trends (data not shown). Therefore, we report only 100-df time-trend smoothers for joint pollutant analyses in this study.

*O_3_.* There are several fairly recent meta-analyses (Bell et al. 2005; Ito et al. 2005; Levy et al. 2005) and multicity analyses (Bell et al. 2004; Katsouyanni and Samet 2009; Smith et al. 2009) of the association between O_3_ and daily deaths. As Smith et al. (2009) point out, there are two kinds of potential biases in meta-analyses: publication bias and model selection bias (in which the investigators test many models and report the results of only those models that show positive and significant associations). Therefore, multicity analyses are preferable to meta-analyses.

The first multicity analyses were conducted by Dominici et al. (2003). These analyses were based on 8 years of data in 90 cities in the United States. For a 10-ppb increase in daily O_3_, Dominici et al. (2003) reported a national mean increase of approximately 0.25% in deaths on the following day, and this estimate was statistically significant. In an update of these analyses in 95 cities and with 14 years of data, Bell et al. (2004) reported a statistically significant national mean of approximately 0.2% (approximate 95% posterior interval: 0.1, 0.3%) for a 1-day lag O_3_ effect on mortality. In a reanalysis of data from the NMMAPS (Katsouyanni and Samet 2009), the O_3_ mortality estimates were highly sensitive to the degree and type of smoothing used for seasonality control, and inclusion of PM_10_ markedly reduced the O_3_ mortality estimates. Our estimated mean with 100 df smoothers for time trends is 0.40% (95% CI: 0.27, 0.56). With 50 df smoothers for time trends, the estimated mean for O_3_ is 0.08% (95% CI: –0.16, 0.38). Thus, the estimated O_3_ effect is sensitive to the degree of smoothing of temporal trends, in agreement with the results reported by Katsouyanni and Samet (2009). Because O_3_ is a highly seasonal pollutant, adequate control of seasonality is important, and using only 50 df for time trends may not be sufficient. To investigate this issue more thoroughly, further analyses of the data stratified by season should be undertaken.

In joint pollutant analyses (100 df smoothers for time trends) with PM_10_, the O_3_ coefficient is substantially attenuated and becomes insignificant, as also reported by Smith et al. (2009) and Katsouyanni and Samet (2009). One possible reason for the widening of CIs for O_3_ effects is the greatly reduced data set used in joint pollutant analyses. If this were the sole reason, however, one would expect to observe the same phenomenon for the PM_10_ effect, which was virtually unchanged and continued to remain highly significant.

In an update to the Bell et al. (2004) study, Bell et al. (2006) examined the ambient concentration–response curve for O_3_ and mortality, where their O_3_ metric was the average of the 24-hr mean concentrations on the current and previous days. Among various approaches evaluating ambient concentration–response relationships, Bell et al. (2006) used natural splines followed by a hierarchical Bayes procedure to obtain a national estimate. The results shown in Figure 3 of their article suggest the possibility of a threshold at about 15 ppb. Smith et al. (2009) fit a piece-wise linear ambient concentration–response relationship with breaks at 40 ppb and 60 ppb, and they showed that a hockey-stick–shaped curve with a break at 40 ppb is consistent with the data. Our analysis shows evidence of a threshold at a little over 30 ppb (Figure 1B). However, the data are too sparse to draw any firm conclusions regarding the shape of the concentration–response curve at low concentrations. We emphasize here that our analyses are based on 24-hr average concentrations of O_3_, whereas regulation is based on the maximum 8-hr averages. Pollutant effect estimates cannot easily be converted from one averaging time to another. Smith et al. (2009) showed that a simple scaling procedure is inadequate. It is, therefore, important to repeat these analyses with other measures of exposure.

*CO, NO_2_, SO_2_*. The only previous multicity analyses of these three pollutants were conducted during reanalyses of NMMAPS (Dominici et al. 2003). For increases of 1 ppm for CO and 10 ppb for NO_2_ and SO_2_, respectively, the NMMAPS investigators reported statistically significant increases in all-cause mortality at a 1-day lag of approximately 0.5%, 0.25%, and 0.6%. Our single-pollutant estimates of the mean are higher for each of these pollutants (e.g., 1.3%, 1.0%, and 1.5%, respectively, using 100 df splines for time trends). In a multipollutant model including these three gases, CO was no longer significantly associated with mortality (mean 0.64%; 95% CI: –0.20, 1.09), but NO_2_ and SO_2_ remained so (mean 0.62; 95% CI: 0.40, 0.98 and mean 0.82; 95% CI: 0.48, 1.15, respectively). In interpreting this result it should be kept in mind that, because of missing data, multipollutant analyses are based on a smaller set of cities than single-pollutant analyses. Concentration–response relationships for CO and SO_2_ are consistent with linearity. Although there is the suggestion of a threshold at around 20 ppb for NO_2_, as for PM_10_, the confidence bounds are too wide to rule out linearity.

## Conclusions

We used the subsampling bootstrap procedure to derive maximum likelihood estimators of national effects of exposures to criteria pollutants on deaths the following day. The AIC for the fitted models provided little evidence of a common effect estimate across the United States, however (data not shown). While the focus of much air pollution research for the past couple of decades has been on PM and O_3_, we find stronger associations between CO, NO_2_, and SO_2_ and mortality. The ambient concentration–response relationship for O_3_ shows evidence of nonlinearity. Regional and seasonal analyses using the methods described in this study may offer further insight. Previous publications have reported that the results of time-series analyses of air pollution data in individual cities can be highly sensitive to choice of statistical model (e.g., Clyde 2000; Koop and Tole 2004; Moolgavkar 2003). While our analyses are based on the most recent data available to us (which are not identical to the data used in previous analyses), our results suggest that different statistical approaches to multicity analyses can yield disparate results.
